# Measuring Integrated Information from the Decoding Perspective

**DOI:** 10.1371/journal.pcbi.1004654

**Published:** 2016-01-21

**Authors:** Masafumi Oizumi, Shun-ichi Amari, Toru Yanagawa, Naotaka Fujii, Naotsugu Tsuchiya

**Affiliations:** 1 RIKEN Brain Science Institute, Wako, Saitama, Japan; 2 School of Psychological Sciences, Faculty of Biomedical and Psychological Sciences, Monash University, Clayton, Victoria, Australia; 3 Japan Science and Technology Agency, Kawaguchi, Saitama, Japan; 4 Monash Institute of Cognitive and Clinical Neurosciences, Monash University, Clayton, Victoria, Australia; University of Hertfordshire, UNITED KINGDOM

## Abstract

Accumulating evidence indicates that the capacity to integrate information in the brain is a prerequisite for consciousness. Integrated Information Theory (IIT) of consciousness provides a mathematical approach to quantifying the information integrated in a system, called integrated information, Φ. Integrated information is defined theoretically as the amount of information a system generates as a whole, above and beyond the amount of information its parts independently generate. IIT predicts that the amount of integrated information in the brain should reflect levels of consciousness. Empirical evaluation of this theory requires computing integrated information from neural data acquired from experiments, although difficulties with using the original measure Φ precludes such computations. Although some practical measures have been previously proposed, we found that these measures fail to satisfy the theoretical requirements as a measure of integrated information. Measures of integrated information should satisfy the lower and upper bounds as follows: The lower bound of integrated information should be 0 and is equal to 0 when the system does not generate information (no information) or when the system comprises independent parts (no integration). The upper bound of integrated information is the amount of information generated by the whole system. Here we derive the novel practical measure Φ* by introducing a concept of mismatched decoding developed from information theory. We show that Φ* is properly bounded from below and above, as required, as a measure of integrated information. We derive the analytical expression of Φ* under the Gaussian assumption, which makes it readily applicable to experimental data. Our novel measure Φ* can generally be used as a measure of integrated information in research on consciousness, and also as a tool for network analysis on diverse areas of biology.

## Author Summary

Integrated Information Theory (IIT) of consciousness attracts scientists who investigate consciousness owing to its explanatory and predictive powers for understanding the neural properties of consciousness. IIT predicts that the levels of consciousness are related to the quantity of information integrated in the brain, which is called integrated information Φ. Integrated information measures excess information generated by a system as a whole above and beyond the amount of information independently generated by its parts. Although IIT predictions are indirectly supported by numerous experiments, validation is required through quantifying integrated information directly from experimental neural data. Practical difficulties account for the absence of direct, quantitative support. To resolve these difficulties, several practical measures of integrated information have been proposed. However, we found that these measures do not satisfy the theoretical requirements of integrated information: First, integrated information should not be below 0; and second, integrated information should not exceed the quantity of information generated by the whole system. Here, we propose a novel practical measure of integrated information, designated as Φ* that satisfies these theoretical requirements by introducing the concept of mismatched decoding developed from information theory. Φ* creates the possibility of empirical and quantitative validations of IIT to gain novel insights into the neural basis of consciousness.

## Introduction

Although its neurobiological basis remains unclear, consciousness may be related to certain aspects of information processing [1, 2]. In particular, Integrated Information Theory of consciousness (IIT) developed by Tononi and colleagues [2–9] predicts that the amount of information integrated among the components of a system, called integrated information Φ, is related to the level of consciousness of the system. The level of consciousness in the brain varies from a very high level, as in full wakefulness, to a very low level, as in deeply anesthetized states or dreamless sleep. When consciousness changes from high to low, IIT predicts that the amount of integrated information changes from high to low, accordingly. This prediction is indirectly supported by recent neuroimaging experiments that combine noninvasive magnetic stimulation of the brain (transcranial magnetic stimulation, TMS) with electrophysiological recordings of stimulation-evoked activity (electroencephalography) [10–14]. Such evidence implies that if there is a practical method to estimate the amount of integrated information from neural activities, we may be able to measure levels of consciousness using integrated information.

IIT provides several versions of mathematical formulations to calculate integrated information [2–8]. Although the detailed mathematical formulations are different, the central philosophy of integrated information does not vary among different versions of IIT. Integrated information is mathematically defined as the amount of information generated by a system as a whole above and beyond the amount of information generated independently by its parts. If the parts are independent, no integrated information should exist.

Despite its potential importance, the empirical calculation of integrated information is difficult. For example, one difficulty involves making an assumption when integrated information is calculated according to the informational relationship between the past and present states of a system. The distribution of the past states is assumed to maximize entropy, which is called the maximum entropy distribution. The assumption of the maximum entropy distribution severely limits the applicability of the original integrated information measure Φ as indicated by [15]. First, the concept of the maximum entropy distribution cannot be applied to a system that comprises elements whose states are continuous, because there is no unique maximum entropy distribution for continuous variables [15, 16]. Second, information under the assumption of the maximum entropy distribution can be computed only when there is complete knowledge about the transition probability matrix that describes how the system transits between states. However, the transition probability matrix for actual neuronal systems is practically impossible to estimate.

To overcome these problems, Barrett and Seth [15] proposed using the empirical distribution estimated from experimental data, thereby removing the requirement to rely on the assumption of the maximum entropy distribution. Although we believe that their approach does lead to practical computation of integrated information, we found that their proposed measures based on the empirical distribution [15] do not satisfy key theoretical requirements as a measure of integrated information. Two theoretical requirements should be satisfied as a measure of integrated information. First, the amount of integrated information should not be negative. Second, the amount of integrated information should never exceed information generated by the whole system. These theoretical requirements, which are satisfied by the original measure Φ, are required so that a measure of integrated information is interpretable in accordance with the original philosophy of integrated information.

Here, we propose a novel practical measure of integrated information, Φ*, by introducing the concept of mismatched decoding developed from information theory [17–20]. Φ* represents the difference between “actual” and “hypothetical” mutual information between the past and present states of the system. The actual mutual information corresponds to the amount of information that can be extracted about the past states by knowing the present states (or vice versa) when the actual probability distribution of a system is used for decoding. In contrast, hypothetical mutual information corresponds to the amount of information that can be extracted about the past states by knowing the present states when the “mismatched” probability distribution is used for decoding where a system is partitioned into hypothetical independent parts. Decoding with a mismatched probability distribution is called mismatched decoding. Φ* quantifies the amount of loss of information caused by the mismatched decoding where interactions between the parts are ignored. We show here that Φ* satisfies the theoretical requirements as a measure of integrated information. Further, we derive the analytical expression of Φ* under the Gaussian assumption and make this measure feasible for practical computation. We also compute Φ* and the previously proposed measures in electrocorticogram (ECoG) data recorded in monkeys to demonstrate that the previous measures violate the theoretical requirements even in real brain recordings.

## Results

While its central ideas are unchanged, IIT updated measures of integrated information. The original formulation, IIT 1.0 [2], underwent major developments leading to IIT 2.0 [6] and the latest version IIT 3.0 [8]. In the present study, we focus on the version in IIT 2.0 [3, 6], because the measure of integrated information proposed in IIT 2.0 is simpler and more feasible to calculate compared with that in IIT 3.0 [5, 8].

Here, we briefly review the original measure of integrated information, Φ, in IIT 2.0 [3, 6] and describe its limitations for practical application [15]. From the concept of the original measure, we point out the lower and upper bounds that a measure of integrated information should satisfy. We introduce next two practical measures of integrated information, Φ_*I*_ and Φ_*H*_, proposed by [15] and show that Φ_*I*_ and Φ_*H*_ fail to satisfy the lower and upper bounds of integrated information. Finally, we derive a novel measure of integrated information, Φ*, from the decoding perspective, which is properly bounded from below and above.

### Intrinsic information and extrinsic information

In IIT, information refers to intrinsic information as opposed to extrinsic information (See S1 Text for details). Intrinsic information is quantified from the intrinsic perspective of a system itself and only depends on internal variables of the system. On the other hand, extrinsic information is quantified from the extrinsic perspective of an external observer and depends on external variables. For example, in neuroscience, extrinsic information is quantified as mutual information between neural states *X* and external stimuli *S*, *I*(*X*;*S*) [21–24]. In contrast, intrinsic information can be quantified by the mutual information between the past states *X* ^*t*−*τ*^ and the present states *X* ^*t*^ of the system, *I*(*X* ^*t*−*τ*^;*X* ^*t*^). The mutual information, *I*(*X* ^*t*−*τ*^;*X* ^*t*^), is expressed by

$$I\;\left(X^{t-\tau };X^{t}\right)=H\;\left(X^{t-\tau }\right)-H\;\left(X^{t-\tau }|X^{t}\right),$$ (1)

where *H*(*X* ^*t*−*τ*^) is the entropy of the past states and *H*(*X* ^*t*−*τ*^|*X* ^*t*^) is the conditional entropy of the past states given the present states. In IIT, the distribution of the past states is assumed to be the maximum entropy distribution so that the entropy of the past states is maximized, i.e., the past states are maximally uncertain. We can interpret that intrinsic information, *I*(*X* ^*t*−*τ*^;*X* ^*t*^), quantifies to what extent uncertainty of the past states can be reduced by knowing the present states from the system’s intrinsic point of view. IIT considers such quantity as the amount of information intrinsically generated by the system.

### Measure of integrated information with the maximum entropy distribution

Consider partitioning a system into *m* parts such as *M*_1_, *M*_2_, ⋯, and *M*_*m*_ and computing the quantity of information that is integrated across the *m* parts of a system. As detailed in S1 Text, the measure of integrated information proposed in IIT 2.0 can be expressed as follows:

$$\Phi =I\;{(}^{\max}X^{t-\tau };X^{t})-\sum_{i=1}^{m}I\;{(}^{\max}M_{i}^{t-\tau };M_{i}^{t}),$$ (2)

where the superscript^max^ indicates that the distribution of the past states is the maximum entropy distribution. The first term of Eq 2, *I*(^max^ *X* ^*t*−*τ*^;*X* ^*t*^), represents the mutual information between the past and present states in the whole system, and the second term represents the sum of the mutual information between the past and present states in the *i*-th part of the system $I{(}^{\max}M_{i}^{t-\tau };M_{i}^{t})$. Thus, Φ, the difference between them, gives the information generated by the whole system above and beyond the information generated independently by its parts. If the parts are independent, no extra information is generated, and the integrated information is 0. We can rewrite Eq 2 in terms of entropy *H* as follows:

$$\Phi =\sum_{i=1}^{m}H\;{(}^{\max}M_{i}^{t-\tau }|M_{i}^{t})-H\;{(}^{\max}X^{t-\tau }|X^{t}).$$ (3)

To derive the above expression, we use the fact that the entropy of the whole system *H*(^max^ *X* ^*t*−*τ*^) equals the sum of the entropy of the subsystems $\sum_{i=1}^{m}H{(}^{\max}M_{i}^{t-\tau })$ when the maximum entropy distribution is assumed.

### Theoretical requirements as a measure of integrated information

To interpret a measure of integrated information as the “extra” information generated by a system as a whole above and beyond its parts, it should satisfy theoretical requirements, as follows: First, integrated information should not be negative because information independently generated by the parts should never exceed information generated by the whole. Integrated information should equal 0 when the amount of information generated by the whole system equals 0 (no information) or when the amount of information generated by the whole is equal to that generated by its parts (no integration). Second, integrated information should not exceed the amount of information generated by the whole system because the information generated by the parts should not be negative. In short, integrated information should be lower-bounded by 0 and upper-bounded by the information generated by the whole system.

One can check the original measure Φ satisfies the lower and upper bounds.

$$0\leq \Phi \leq I\;{(}^{\max}X^{t-\tau };X^{t}).$$ (4)

As shown in S1 Text, Φ can be written as the Kullback-Leibler divergence. Thus, Φ is positive or equal to 0. Further, as can be seen from Eq 2, the upper bound of Φ is the mutual information in the entire system, because the sum of mutual information in the parts is larger than or equal to 0.

#### Practical measures of integrated information with empirical distribution

The original measure Φ assumes the distribution of the past states to be the maximum entropy distribution, which limits the practical application of Φ for two reasons. First, the maximum entropy distribution can be applied only when the states of a system are discrete. If the states are represented by discrete variables, the maximum entropy distribution is the uniform distribution over all possible states of *X* ^*t*−*τ*^. When the states of a system are described by continuous variables, the maximum entropy distribution cannot be uniquely defined [15, 16]. Second, the transition probability matrix of a system, *p*(*X* ^*t*^|*X* ^*t*−*τ*^) must be known for all possible past states *X* ^*t*−*τ*^ for obtaining the mutual information *I*(^max^ *X* ^*t*−*τ*^;*X* ^*t*^). However, it is nearly impossible to estimate such a complete transition probability matrix experimentally in an actual neural system, because some states may not occur during a reasonable period of observation.

A simple remedy for the limitations of the original measure Φ is not to impose the maximum entropy distribution on the past states but instead to use the probability distributions obtained from empirical observations of the system. Barrett and Seth [15] adopted this strategy to derive two practical measures of integrated information from Eqs 2 and 3 by substituting the maximum entropy distribution with the empirical distribution as follows:

$$\Phi_{I}=I\;\left(X^{t-\tau };X^{t}\right)-\sum_{i=1}^{m}I\;\left(M_{i}^{t-\tau };M_{i}^{t}\right),$$ (5)

$$\Phi_{H}=\sum_{i=1}^{m}H\;\left(M_{i}^{t-\tau }|M_{i}^{t}\right)-H\;\left(X^{t-\tau }|X^{t}\right).$$ (6)

Note that Φ_*I*_ and Φ_*H*_ are not equal when the empirical distribution is used for the past states, because the entropy of the whole system *H*(*X* ^*t*−*τ*^) is not equal to the sum of the entropy of the subsystems, $\sum_{i}H\left(M_{i}^{t-\tau }\right)$. Φ_*H*_ was also derived from a different perspective from IIT, i.e. the perspective of information geometry, as a measure of spatio-temporal interdependencies and is termed “stochastic interaction” [25, 26].

Although these two measures appear as natural modifications of the original measure, they do not satisfy the theoretical requirements as a measure of integrated information. We discuss the problems of Φ_*I*_ and Φ_*H*_ in detail later.

#### Integrated information measure based on mismatched decoding

**Fig 1:**
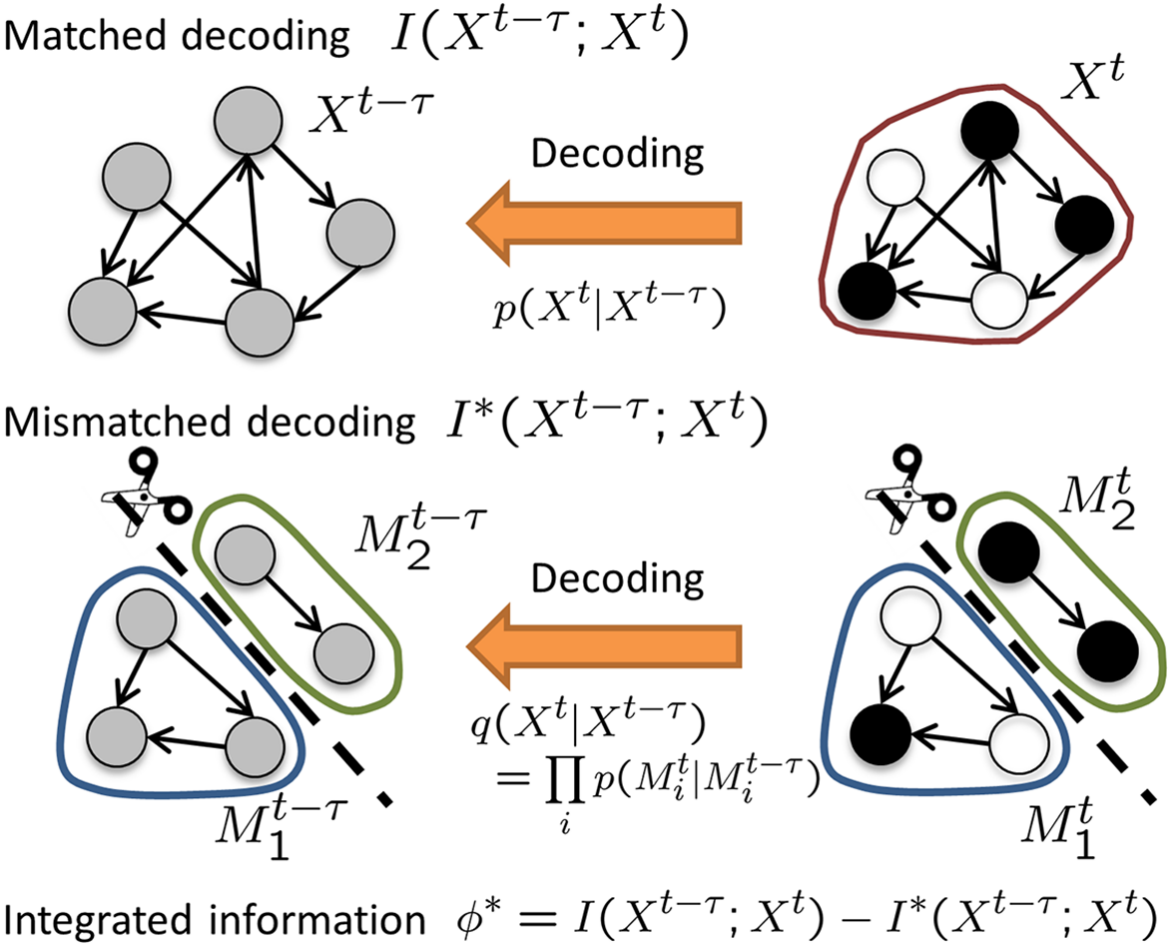
Integrated information based on the concept of mismatched decoding. The figure shows a system with five neurons in which the arrows represent directed connectivity and the colors represent the states of the neurons (black: silence, white: firing, gray: unknown). The past states *X* ^*t*−*τ*^ are decoded given the present states *X* ^*t*^. The “true” conditional distribution *p*(*X* ^*t*^|*X* ^*t*−*τ*^) is used for matched decoding, while a “false” conditional distribution *q*(*X* ^*t*^|*X* ^*t*−*τ*^) is used for mismatched decoding where the parts of a system *M*_1_ and *M*_2_ are assumed independent. The amount of information about the past states that can be extracted from the present states using matched and mismatched decoding is quantified by the mutual information *I*(*X* ^*t*−*τ*^;*X* ^*t*^) and the “hypothetical” mutual information I*(*X* ^*t*−*τ*^;*X* ^*t*^) for mismatched decoding, respectively. In this framework, integrated information, Φ*(*X* ^*t*−*τ*^;*X* ^*t*^), is defined as the difference between *I*(*X* ^*t*−*τ*^;*X* ^*t*^) and I*(*X* ^*t*−*τ*^;*X* ^*t*^).

Here, we propose an alternative practical measure of integrated information that satisfies the theoretical requirements which we call Φ* (phi star) (Fig 1). Φ*, which uses the empirical distribution, can be applied to actual neuronal recordings. Similar to Φ_*I*_, we will derive Φ* based on the original measure Φ in Eq 2 based on mutual information. Given the problem of Φ_*I*_ in Eq 5, we should refine the second term of Eq 5, while the first term, the mutual information in the whole system, is unchanged. The second term should be a quantity that can be interpreted as information generated independently by the parts of a system and should be less than information generated by the system as a whole.

To derive a proper second term in Eq 5, we interpret the mutual information from a decoding perspective and introduce the concept of “mismatched decoding”, which was developed by information theory [17] (see S1 Text for details). Consider that the past states *X* ^*t*−*τ*^ are decoded given the present states *X* ^*t*^. From the decoding perspective, the mutual information can be interpreted as the maximum information about the past states that can be obtained knowing the present states. To extract the maximum information, the decoding must be performed optimally using the “true” conditional distribution,

$$p\left(X^{t}|X^{t-\tau }\right)=p\left(M_{1}^{t},\cdots ,M_{m}^{t}|M_{1}^{t-\tau },\cdots ,M_{m}^{t-\tau }\right).$$ (7)

Note that the expression on the right explicitly accounts for interactions among all the parts. The optimal decoding can be performed using the maximum likelihood estimation. In the above setting, the maximum likelihood estimation chooses the past state that maximizes *p*(*X* ^*t*^|*X* ^*t*−*τ*^) given a present state. Decoding that uses the true distribution, *p*(*X* ^*t*^|*X* ^*t*−*τ*^), is called “matched decoding” because the probability distribution used for decoding matches the actual probability distribution.

Decoding that uses a “false” conditional distribution, *q*(*X* ^*t*^|*X* ^*t*−*τ*^), is called “mismatched” decoding. To quantify integrated information, we consider specifically the mismatched decoding that uses the “partitioned” probability distribution *q*(*X* ^*t*^|*X* ^*t*−*τ*^),

$$q\left(X^{t}|X^{t-\tau }\right)=\prod_{i=1}^{m}p\left(M_{i}^{t}|M_{i}^{t-\tau }\right),$$ (8)

where a system is partitioned into parts and the parts *M*_*i*_ are assumed to be independent. *q*(*X* ^*t*^|*X* ^*t*−*τ*^) is the product of the conditional probability distribution in each part $p(M_{i}^{t}|M_{i}^{t-\tau })$. The distribution, *q*(*X* ^*t*^|*X* ^*t*−*τ*^), is “mismatched” with the actual probability distribution, because parts are generally not independent in reality. As is matched decoding, mismatched decoding is also performed using the maximum likelihood estimation, wherein the past state that maximizes *q*(*X* ^*t*^|*X* ^*t*−*τ*^) is selected. The amount of information obtained from mismatched decoding is necessarily degraded compared with that obtained from matched decoding. The best decoding performance can be achieved only when matched decoding is used with the actual probability distribution *p*(*X* ^*t*^|*X* ^*t*−*τ*^).

We consider the amount of information that can be obtained from mismatched decoding, I*(*X* ^*t*−*τ*^;*X* ^*t*^), as a proper second term of Eq 5 (see Methods for the mathematical expression of I*). The difference between *I*(*X* ^*t*−*τ*^;*X* ^*t*^) and I*(*X* ^*t*−*τ*^;*X* ^*t*^) provides a new practical measure of integrated information (Fig 1),

$$\Phi^{*}\left(X^{t-\tau };X^{t}\right)=I\;\left(X^{t-\tau };X^{t}\right)-I^{*}\;\left(X^{t-\tau };X^{t}\right).$$ (9)

Φ* quantifies the information loss caused by mismatched decoding where a system is partitioned into independent parts, and the interactions between the parts are ignored. Φ* satisfies the theoretical requirements, because I* is greater than or equal to 0 and is less than or equal to the information in the whole system *I*. Φ* is equivalent to the original measure Φ if the maximum entropy distribution is imposed on the past states instead of an empirical distribution (see S1 Text for the proof). Thus, we can consider Φ* as a natural extension of Φ to the case when the empirical distribution is used.

### Analytical computation of Φ* using Gaussian approximation

Although using an empirical distribution instead of the maximum entropy distribution makes integrated information feasible to calculate, it is still difficult to compute Φ* in a large system, because the summation over all possible states must be calculated. The number of all possible states grows exponentially with the size of the system and therefore, computational costs for computing Φ* also grow exponentially. Thus, for practical calculation of Φ*, we need to approximate Φ* in some way such as approximating the probability distribution of neural states using the Gaussian distribution [15]. Φ* can be analytically computed using the Gaussian approximation (see Methods). The Gaussian approximation significantly reduces the computational costs and makes Φ* practically computable even in a large system.

### Theoretical requirements are not satisfied by previously proposed measures

In this section, by considering two extreme cases, we demonstrate that the previously proposed measures Φ_*H*_ and Φ_*I*_[15] do not satisfy either the lower or upper bound.

#### When there is no information

First, we consider the case where there is no information between the past and present states of a system, i.e. *I*(*X* ^*t*−*τ*^;*X* ^*t*^) = 0. In this case, integrated information should be 0. As expected, Φ* and Φ_*I*_ are 0, because the amount of information for mismatched decoding, I*(*X* ^*t*−*τ*^;*X* ^*t*^), and the mutual information in each part, $I(M_{i}^{t-\tau };M_{i}^{t})$, are both 0 when *I*(*X* ^*t*−*τ*^;*X* ^*t*^) = 0;

$$\Phi^{*}=0,$$ (10)

$$\Phi_{I}=0.$$ (11)

However, Φ_*H*_ is not 0. Φ_*H*_ can be written as

$$\Phi_{H}=\sum_{i}H\;\left(M_{i}^{t-\tau }\right)-H\;\left(X^{t-\tau }\right).$$ (12)

Φ_*H*_ is not 0 even when the information *I*(*X* ^*t*−*τ*^;*X* ^*t*^) is 0 because Φ_*H*_ is not based on the mutual information but on the conditional entropy (see Eq 6). Therefore, Φ_*H*_ does not necessarily reflect the amount of information in a system.

As a simple example that shows the above problem of Φ_*H*_, consider the following linear regression model,

$$X^{t}=AX^{t-1}+E^{t}.$$ (13)

Here, *X* is the state of units, *A* is a connectivity matrix, and *E* ^*t*^ is multivariate Gaussian noise with zero mean and covariance Σ(*E*). *E* ^*t*^ is uncorrelated over time. For simplicity, consider a system composed of two units (the following argument can be easily generalized to a system with more than two units). We set the connectivity matrix *A* and the covariance matrix of noise Σ(*E*) as follows:

$$A=a\;\left(\begin{array}{cc} 1 & 1\\ 1 & 1 \end{array}\right),$$ (14)

$$\Sigma \;\left(E\right)=\left(\begin{array}{cc} 1 & c\\ c & 1 \end{array}\right),$$ (15)

where *a* and *c* are parameters that control the strengths of connections and noise correlation, respectively. We compute measures of integrated information using the above model. The time difference *τ* is set to 1. We assume that the prior distribution of the system is the steady state distribution, where the covariance of the past states, Σ(*X* ^*t*−1^), and that of the present states, Σ(*X* ^*t*^), are equal, i.e. Σ(*X* ^*t*−1^) = Σ(*X* ^*t*^) = Σ(*X*). The covariance of the steady state distribution Σ(*X*) can be calculated by taking the covariance of both sides of Eq 13,

$$\Sigma \left(X\right)=A\Sigma \left(X\right)A^{T}+\Sigma \left(E\right).$$ (16)

**Fig 2:**
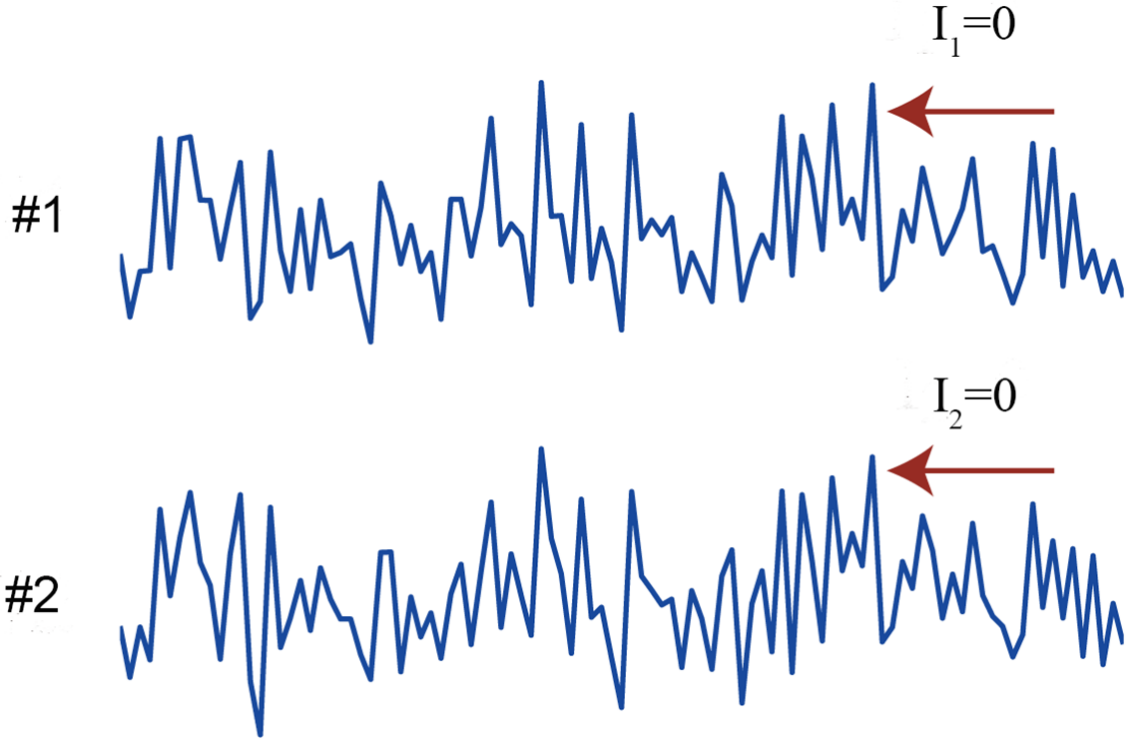
Exemplar time series when there is no information between the past and present states. The connection strength *a* and the strength of noise correlation *c* are set to 0 and 0.9, respectively in the linear regression model (Eq 13). *I*_1_ and *I*_2_ represent the mutual information in units 1 and 2. Because there is no connection, there is no information between the past and present states of the system: *I*_1_ and *I*_2_ are both 0. In this case, Φ* and Φ_*I*_ are 0 as they should be, yet Φ_*H*_ is positive.

We consider a case where the connection strength *a* is 0. Fig 2 shows an exemplar time series when the strength of noise correlation *c* is 0.9. Because there are no connections, including self-connections within each unit, each unit has no information between the past and present states, i.e., *I*_1_ = *I*_2_ = 0. As can be seen from Fig 2, however, the two time series correlate at each moment because of the high noise correlation.

**Fig 3:**
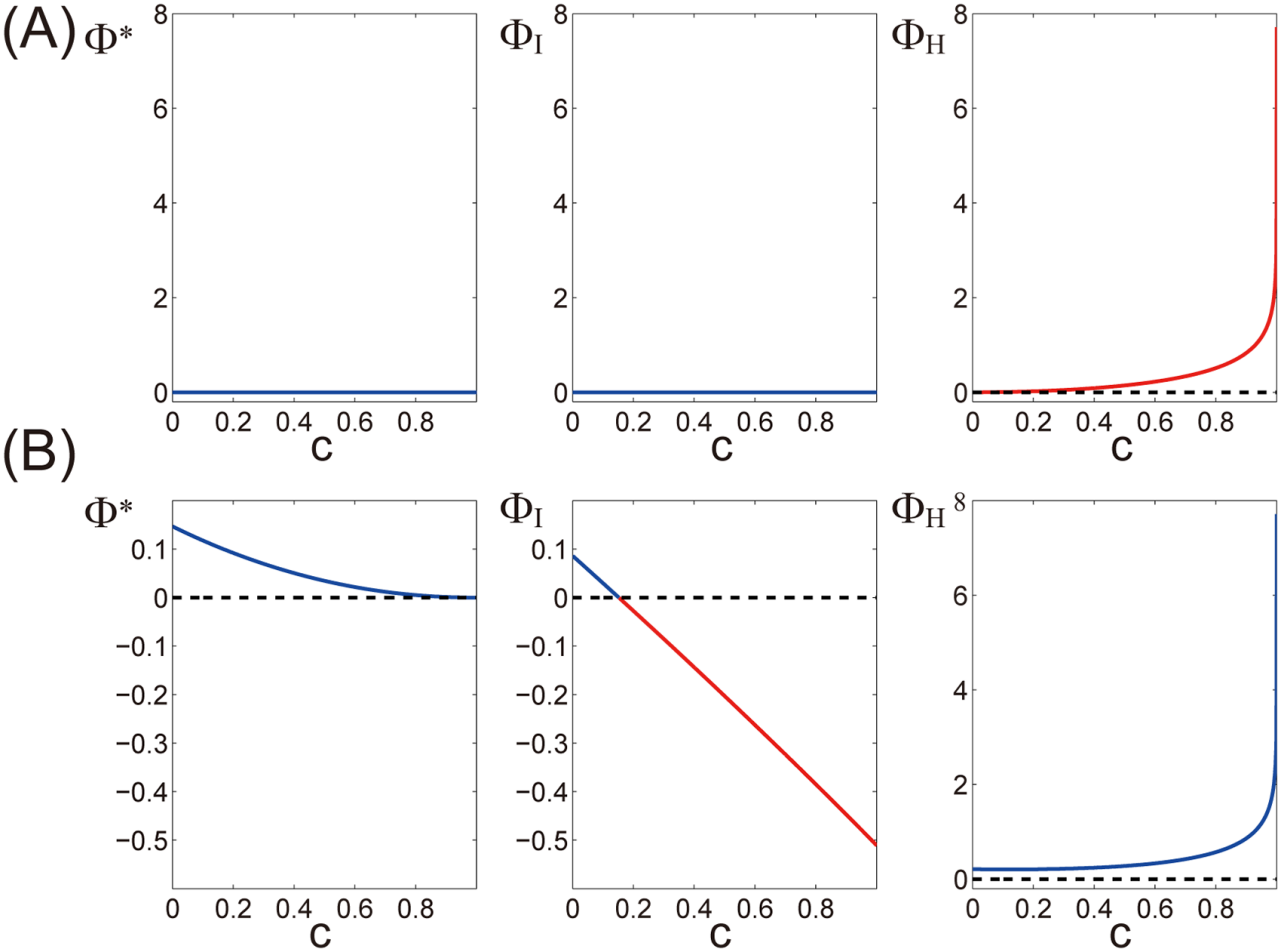
Violation of theoretical requirements as a measure of integrated information. The behaviors of Φ*, Φ_*I*_, and Φ_*H*_ are shown in the left, middle, and right panels, respectively, when the strength of noise correlation *c* is varied in a linear regression model (Eq 13). Red lines indicate the regime where the theoretical requirements are violated, and the blue lines indicate that the theoretical requirements are satisfied. Dotted black lines are drawn at 0. (A) Violation of the upper bound. The strength of connections *a* is set to 0. In this case, there is no information between the past and present states of the system but Φ_*H*_ is not 0, i.e., Φ_*H*_ violates the upper bound. (B) Violation of the lower bound. The strength of connections *a* is set to 0.4. At the right ends of the figures where *c* is 1, the two units in the system are perfectly correlated. Φ_*I*_ is negative, i.e., Φ_*I*_ violates the lower bound when the degree of correlation is high.

We varied the degree of noise correlation, *c*, from 0 to 1 while keeping the connection strength *a* as 0 (Fig 3(A)). Φ* and Φ_*I*_ stay 0 independent of noise correlation. However, an entropy-based measure, Φ_*H*_, increases monotonically with *c*, irrespective of the amount of information in the whole system (Fig 3(A)). As shown in Eq 12, Φ_*H*_ is the difference between the sum of entropy within each part and entropy in the whole system. When the parts correlate, the entropy in the whole system decreases. In contrast, the sum of entropy of each part does not change, because the degree of noise within each part (the diagonal elements of *E* ^*t*^) is fixed. Thus, Φ_*H*_ increases as the degree of noise correlation *c* increases without reflecting the amount of information in the system.

#### When parts are perfectly correlated

Next, we consider the case where the parts are perfectly correlated. More specifically, consider the case where the two parts *M*_1_ and *M*_2_ are equal at every time, i.e. $M_{1}^{t-\tau }=M_{2}^{t-\tau }=M^{t-\tau }$ and $M_{1}^{t}=M_{2}^{t}=M^{t}$. Here, Φ* is 0 because the amount of information extracted by mismatched decoding would not degrade even if the other part is ignored for decoding (see S1 Text for the mathematical proof).

$$\Phi^{*}=0.$$ (17)

Regarding Φ_*I*_, the mutual information of each part is equal to each other, $I\left(M_{1}^{t-\tau };M_{1}^{t}\right)=I\left(M_{2}^{t-\tau };M_{2}^{t}\right)=I\left(M^{t-\tau };M^{t}\right)$ and the mutual information in the whole system is equal to the mutual information of each part, *I*(*X* ^*t*−*τ*^;*X* ^*t*^) = *I*(*M* ^*t*−*τ*^;*M* ^*t*^). Thus, the second term in Eq 5 is twice the value of the first, and Φ_*I*_ is the negative value of the mutual information in one part,

$$\Phi_{I}=-I\;\left(M^{t-\tau };M^{t}\right).$$ (18)

Thus, Φ_*I*_ does not satisfy the lower bound as a measure of integrated information. Φ_*H*_ is given by

$$\Phi_{H}=H\;\left(X^{t-\tau }|X^{t}\right)-2H\;\left(M^{t-\tau }|M^{t}\right),$$ (19)

which is larger than or equal to 0 (Φ_*H*_ is always larger than or equal to 0 because it can be written as the Kullback-Leibler divergence.).

**Fig 4:**
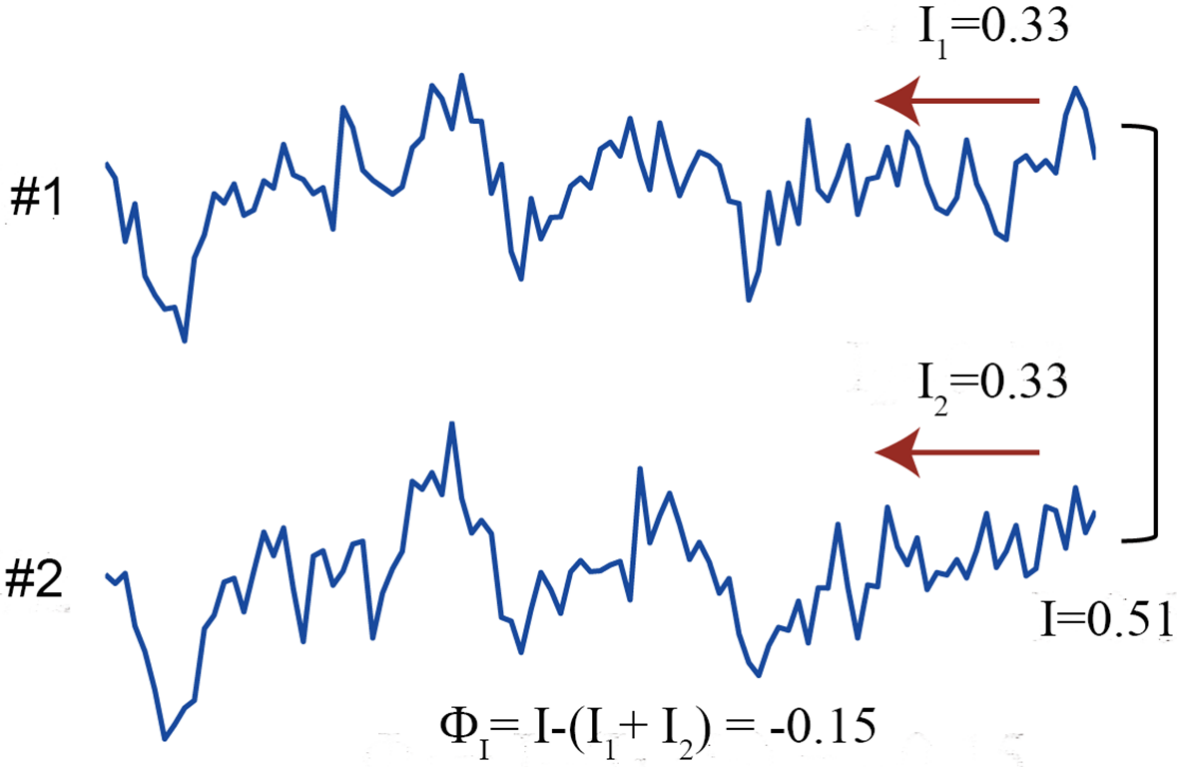
Exemplar time series when correlation is high. The strength of noise correlation *c* and the connection strength *a* are set to both 0.4 in the linear regression model (Eq 13). *I*_1_ and *I*_2_ represent the mutual information in unit 1 and 2, and *I* represents the mutual information in the whole system. In this case, the sum of the mutual information in the parts exceeds the mutual information in the whole system and Φ_*I*_ is negative.

We considered again the same linear regression model presented in the previous section (Eq 13). We varied the degree of noise correlation, *c*, from 0 to 1 while keeping connection strength *a* as 0.4. When *c* is 1, the two units correlate perfectly. Fig 4 shows an exemplar time series when *c* is 0.4 and *a* is 0.4. Φ_*I*_ takes positive values when *c* is less than ∼0.2 but takes negative values when *c* is greater (Fig 3(B)). Φ* decreases monotonically with *c* and becomes 0 when *c* is 1. Φ_*H*_ increases monotonically with *c* reflecting the degree of correlation between the units. The detailed behaviors of Φ*, Φ_*I*_ and Φ_*H*_ when *a* and *c* are both varied are shown in S1 Fig.

#### Electrocorticogram data analysis

**Fig 5:**
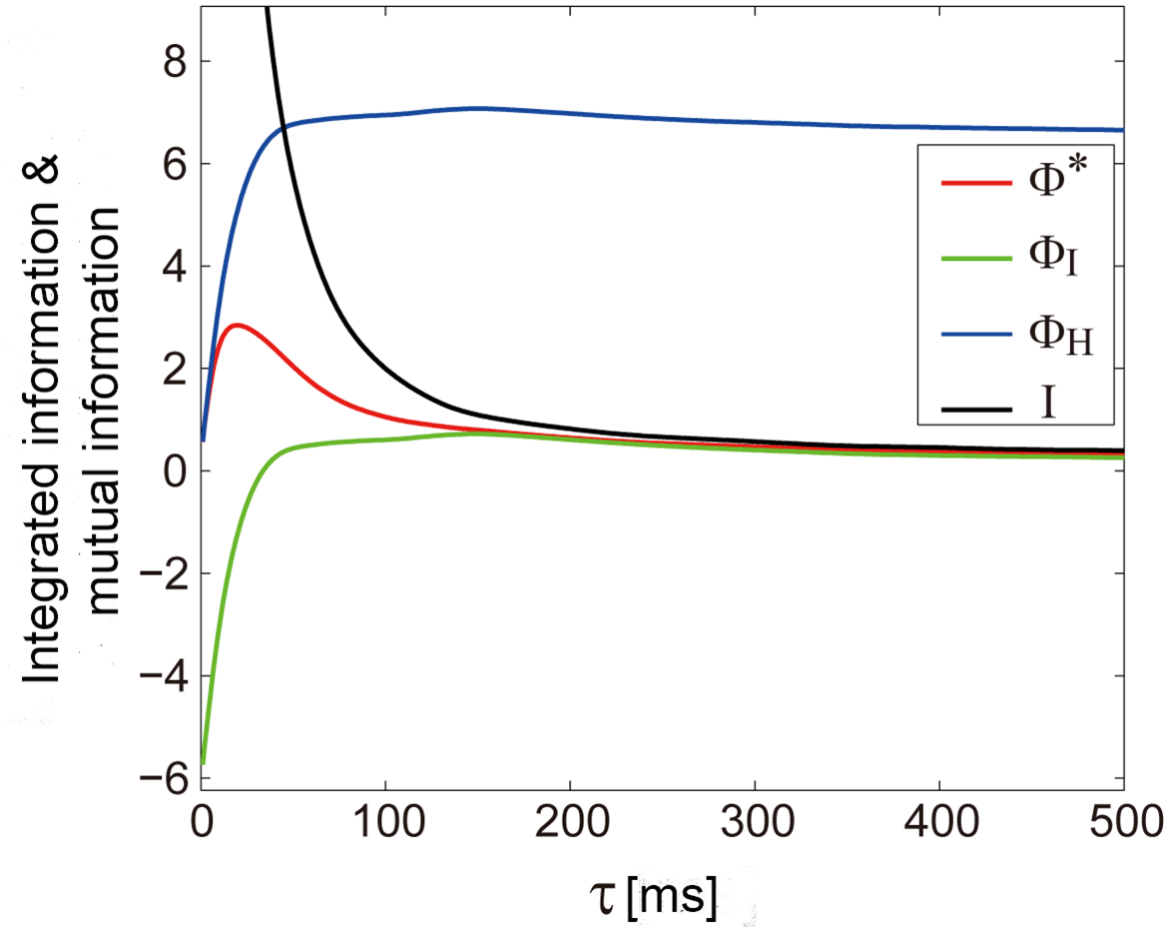
Measures of integrated information and mutual information computed in monkey ECoG data. Time lag *τ* is varied from 1 to 500 ms. The behaviors of Φ* (red line), Φ_*I*_ (green line), Φ_*H*_ (blue line), and mutual information *I* (black line) are shown. Φ_*I*_ and Φ_*H*_ violate the theoretical requirements.

The problems of Φ_*H*_ and Φ_*I*_ can manifest in their application to real neural recordings from the brain. Fig 5 shows the measures of integrated information, Φ*, Φ_*I*_, Φ_*H*_, and the mutual information *I* computed from the electrocorticogram (ECoG) recordings in an awake monkey as a function of the time lag *τ* (See Methods for details).

As we can see, the mutual information between *X* ^*t*^ and *X* ^*t*−*τ*^ monotonically decreases as *τ* increases. Φ* is positive, peaks around *τ* = 20 ms, and less than the mutual information, always satisfying the theoretical requirements. However, Φ_*I*_ is negative when *τ* is small and Φ_*H*_ remains large even when *I* approaches 0 with increasing *τ*, both violating the theoretical requirements.

## Discussion

In this study, we consider the two theoretical requirements that a measure of integrated information should satisfy, as follows: The lower and upper bounds of integrated information should be 0 and the amount of information generated by the whole system, respectively. The theoretical requirements are naturally derived from the original philosophy of integrated information [3, 6], which states that integrated information is the information generated by a system as a whole above and beyond its parts. The original measure of integrated information Φ satisfies the theoretical requirements so that we can interpret a measure of integrated information according to the original philosophy. To derive a practical measure of integrated information that satisfies the required lower and upper bounds, we introduced a concept of mismatched decoding. We defined our measure of integrated information Φ* as the amount of information lost when a mismatched probability distribution, where a system is partitioned into “independent” parts, is used for decoding instead of the actual probability distribution. In this framework, Φ* quantifies the amount of information loss associated with mismatched decoding where interactions between the parts of a system are ignored and therefore quantifies the amount of information integrated by the interactions. We show that Φ* satisfies the lower and upper bounds, that Φ_*I*_ does not satisfy the lower bound, and that Φ_*H*_ does not satisfy the upper bound. We consider Φ* a proper measure of integrated information that can be generally used for practical applications.

Here, we briefly note a potential reason why the previous study [15] failed to identify these problems of Φ_*I*_ and Φ_*H*_. Although they calculated their measures in small networks by using the autoregressive model in Eq 12, they did not extensively vary the connectivity matrix *A* and the Gaussian noise *E*. In particular, they fixed the covariance of the Gaussian noise *E* to 0. As we can clearly see in Fig 3 and S1 Fig, both connectivity strength *a* and the covariance of the noise *c* strongly affect the amount of integrated information. In particular, when the covariance of *E* is large, Φ_*I*_ and Φ_*H*_ violate the theoretical requirements. For future investigations of calculating integrated information in networks described by autoregressive model, we should note that it is very important to take account of not only the effects of connectivity matrix *A* but also the effects of covariance of *E* on the amount of integrated information.

The basic concept of Integrated Information Theory (IIT) was tested by conducting empirical experiments, and the evidence accumulated supports the conclusion that when consciousness is lost, integration of information is lost [10–14]. In particular, Casali and colleagues [14] found that a complexity measure, motivated by IIT, successfully separates conscious awake states from various unconscious states due to deep sleep, anesthesia, and traumatic brain injuries. Although their measure is inspired by the concept of integrated information, it measures the complexity of averaged neural responses to one particular type of external perturbation (e.g. a TMS pulse to a target region) and does not directly measure integrated information.

There are few studies that directly estimate integrated information in the brain [27, 28] using the measure introduced in IIT 1.0 [2] or Φ_*H*_. Our new measure of integrated information, Φ*, will contribute to experiments designed to test whether integrated information is a key to distinguishing conscious states from unconscious states [29–31].

We considered the measure of integrated information proposed in IIT 2.0 [3, 6], because its computations are feasible. There are several updates in the latest version, IIT 3.0 [8]. In IIT 2.0, integrated information is quantified by measuring how the distribution of the past states differs when a present state is given (see S1 Text for details) whereas in IIT 3.0, it is quantified by measuring how the distribution of the past and future states differs when a present state is given. In other words, IIT 2.0 considers only the information flow from the present to the past while IIT 3.0 additionally considers the information flow from the present to the future. Our measure Φ* does not asymmetrically quantify integrated information from the present to the past or from the present to the future, because the mutual information is a symmetric measure for the time points *t* − *τ* and *t*. An unanswered question is how integrated information should be practically calculated taking account of the both directions of information flow, using an empirical distribution.

An unresolved difficulty that impedes practical calculation of integrated information is how to partition a system. In the present study, we considered only the quantification of integrated information when a partition of a system is given. IIT requires that integrated information should be quantified using the partition where information is least integrated, called the minimum information partition (MIP) [3, 6]. To find the MIP, every possible partition must be examined, yet the number of possible partitions grows exponentially with the size of the system. One way to work around this difficulty would be to develop optimization algorithms to quickly find a partition that well approximates the MIP.

Besides the practical problem of finding the MIP, there remains a theoretical problem of how to compare integrated information across different partitions. Integrated information increases as the number of parts gets larger, because more information is lost by partitioning the system. Further, integrated information is expected to be larger in a symmetric partition where a system is partitioned into two parts of equal size than in an asymmetric partition. IIT 2.0 [6] proposes a normalization factor, which considers these issues. However, there might be other possible ways to perform normalization. It is unclear whether there is a reasonable theoretical foundation that adjudicates the best normalization scheme. Moreover, it is unclear if the normalization factor, which is proposed for systems whose states are represented by discrete variables, is appropriate for systems whose states are represented by continuous variables. The normalization factor, which is based on the entropies of the parts of a system, can be negative because entropy can be negative for continuous variables. Thus, we need a different normalization factor when we deal with continuous variables. Further investigations are required to resolve the practical and theoretical issues related to the MIP.

Although we derived Φ*, because we were motivated by IIT and its potential relevance to consciousness, Φ* has unique meaning from the perspective of information theory, which is independent of IIT. Thus, it can be applied to research fields other than research on consciousness [32]. Φ* quantifies the loss of information when interactions or connections between the units in a system are ignored. Thus, Φ* is expected to be related to connectivity measures such as Granger causality [33] or transfer entropy [34]. It will be interesting to clarify mathematical relationships between Φ* and the other connectivity measures. We expect that information geometry [25, 26, 35, 36] plays an important role for studying the properties of these quantities. Here, we indicate only an apparent difference between them as follows: Φ* intends to measure global integrations in a system as a whole, while traditional bivariate measures such as Granger causality or transfer entropy intends to measure local interactions between elements of the system. Consider that we divide a system into parts *A*, *B*, and *C*. Using integrated information, our goal is to quantify the information integrated among *A*, *B*, and *C* as a whole. In contrast, what we quantify using Granger causality or transfer entropy is the influence of *A* on *B*, *B* on *C*, *C* on *A* and the reverse. It is not obvious how a measure of global interactions in the whole system should be defined and derived theoretically from measures of the local interactions. As an example, one possibility is simply summing up all local interactions and considering the sum as a global measure [37]. Yet, more research is required to determine whether such an approach is a valid method to define global interactions [36]. Φ*, in contrast, is not derived from the local interaction measures but is derived directly by comparing the total mutual information in the whole system with hypothetical mutual information when the system is assumed to be partitioned into independent parts. Thus, the interpretation of Φ* is straightforward from an information theoretical viewpoint. Our measure, which we consider a measure of the global interaction, may provide new insights into diverse research subjects as a novel tool for network analysis.

## Methods

### Mathematical expression of I*

The amount of information for mismatched decoding can be evaluated using the following equation,

$$\begin{array}{cc} I^{*}\left(X^{t-\tau };X^{t}\right)=-\sum_{X^{t}}p\left(X^{t}\right)\log\sum_{X^{t-\tau }}p\left(X^{t-\tau }\right)q{\left(X^{t}|X^{t-\tau }\right)}^{\beta } & \\ & +\sum_{X^{t-\tau },X^{t}}p\left(X^{t-\tau },X^{t}\right)\log q{\left(X^{t}|X^{t-\tau }\right)}^{\beta }, \end{array}$$ (20)

where *β* is the value that maximizes I*. The maximization of I* with respect to *β* is performed by differentiating I* and solving the equation, dI*(*β*)/*dβ* = 0. In general, the solution of the equation can be found using the standard gradient ascent method, because I* is a convex function with respect to *β*[17, 18].

For comparison, the mutual information is given by

$$I\;\left(X^{t-\tau };X^{t}\right)=-\sum_{X^{t}}p\left(X^{t}\right)\log p\left(X^{t}\right)+\sum_{X^{t-\tau },X^{t}}p\left(X^{t-\tau },X^{t}\right)\log p\left(X^{t}|X^{t-\tau }\right).$$ (21)

If a mismatched probability distribution *q*(*X* ^*t*^|*X* ^*t*−*τ*^) is replaced by the actual distribution *p*(*X* ^*t*^|*X* ^*t*−*τ*^) in Eq 20, the derivative of I* becomes 0 when *β* = 1. By substituting *q* = *p* and *β* = 1 into Eq 20, one can check that I* is equal to *I* in Eq 21, as it should be. The amount of information for mismatched decoding, I*, was first derived in the field of information theory as an extension of the mutual information in the case of mismatched decoding [17]. I* was first introduced into neuroscience in [18] and was first applied to the analysis of neural data by [19]. However, I* in the prior neuroscience application [18, 19] was quantified between stimuli and neural states, not between the past and present states of a system, as described in the present study.

### Analytical computation of Φ* under the Gaussian assumption

Assume that the probability distribution of neural states X is the Gaussian distribution,

$$p\left(X\right)=\frac{1}{{\left({\left(2\pi \right)}^{N}\left|\Sigma \left(X\right)\right|\right)}^{1/2}}\exp\left(-\frac{1}{2}{\left(X-\bar{X}\right)}^{T}\Sigma {\left(X\right)}^{-1}\left(X-\bar{X}\right)\right).$$ (22)

where *N* is the number of variables in X, $\bar{X}$ is the mean value of X, and Σ(*X*) is the covariance matrix of X. The Gaussian assumption allows us to analytically compute Φ*, which substantially reduces the costs for computing Φ*. When *X* ^*t*−*τ*^ and *X* ^*t*^ are both multivariate Gaussian variables, the mutual information between *X* ^*t*−*τ*^ and *X* ^*t*^, *I*(*X* ^*t*−*τ*^;*X* ^*t*^), can be analytically computed as

$$I\;\left(X^{t-\tau };X^{t}\right)=\frac{1}{2}\log\frac{\left|\Sigma (X^{t-\tau })\right|}{\left|\Sigma (X^{t-\tau }|X^{t})\right|},$$ (23)

where Σ(*X* ^*t*−*τ*^|*X* ^*t*^) is the covariance matrix of the conditional distribution, *p*(*X* ^*t*−*τ*^|*X* ^*t*^), which is expressed as

$$\Sigma \left(X^{t-\tau }|X^{t}\right)=\Sigma \left(X^{t-\tau }\right)-\Sigma \left(X^{t-\tau },X^{t}\right)\Sigma {\left(X^{t}\right)}^{-1}\Sigma {\left(X^{t-\tau },X^{t}\right)}^{T},$$ (24)

where Σ(*X* ^*t*−*τ*^, *X* ^*t*^) is the cross covariance matrix between *X* ^*t*−*τ*^ and *X* ^*t*^, whose element Σ(*X* ^*t*−*τ*^, *X* ^*t*^)_*ij*_ is given by $\text{cov}(X_{i}^{t-\tau },X_{j}^{t})$.

Similarly, we can obtain the analytical expression of I* as follows:

$$I^{*}\left(\beta \right)=\frac{1}{2}\text{Tr}\;\left(\Sigma (X^{t})R\right)+\frac{1}{2}\log\left(|Q||\Sigma (X^{t-\tau })|\right)-\frac{\beta N}{2},$$ (25)

where Tr stands for trace. *Q* and *R* are given by

$$Q=\Sigma {\left(X^{t-\tau }\right)}^{-1}+\beta \Sigma_{D}{\left(X^{t-\tau }\right)}^{-1}\Sigma_{D}{\left(X^{t},X^{t-\tau }\right)}^{T}\Sigma_{D}{\left(X^{t}|X^{t-\tau }\right)}^{-1}\Sigma_{D}\left(X^{t},X^{t-\tau }\right)\Sigma_{D}{\left(X^{t-\tau }\right)}^{-1},$$ (26)

$$\begin{array}{l} R=\beta \Sigma_{D}{\left(X^{t}|X^{t-\tau }\right)}^{-1}\\ -\beta^{2}\Sigma_{D}{\left(X^{t}|X^{t-\tau }\right)}^{-1T}\Sigma_{D}\left(X^{t},X^{t-\tau }\right)\Sigma_{D}{\left(X^{t-\tau }\right)}^{-1}Q^{-1}\Sigma_{D}{\left(X^{t-\tau }\right)}^{-1}\Sigma_{D}{\left(X^{t},X^{t-\tau }\right)}^{T}\Sigma_{D}{\left(X^{t}|X^{t-\tau }\right)}^{-1}, \end{array}$$ (27)

where Σ_*D*_(*X* ^*t*−*τ*^), Σ_*D*_(*X* ^*t*^, *X* ^*t*−*τ*^) and Σ_*D*_(*X* ^*t*^|*X* ^*t*−*τ*^) are diagonal block matrices. Each block matrix is a covariance matrix of each part, $\Sigma (M_{i}^{t-\tau })$, $\Sigma (M_{i}^{t},M_{i}^{t-\tau })$, and $\Sigma (M_{i}^{t}|M_{i}^{t-\tau })$ where *M*_*i*_ is a subsystem. For example, Σ_*D*_(*X* ^*t*−*τ*^) is given by

$$\Sigma_{D}\left(X^{t-\tau }\right)=\left(\begin{array}{cccc} \Sigma (M_{1}^{t-\tau }) & & & \\ & \Sigma (M_{2}^{t-\tau }) & & \text{0}\\ \text{0} & & \ddots & \\ & & & \Sigma (M_{m}^{t-\tau }) \end{array}\right).$$ (28)

The maximization of I* with respect to *β* is performed by solving the equation dI*(*β*)/*dβ* = 0. The derivative of I*(*β*) with respect to *β* is given by

$$\frac{dI^{*}\left(\beta \right)}{d\beta }=\frac{1}{2}\text{Tr}\;\left(\Sigma \left(X^{t}\right)\frac{dR}{d\beta }\right)+\frac{1}{2}\text{Tr}\;\left(Q^{-1}\frac{dQ}{d\beta }\right)-\frac{N}{2},$$ (29)

where

$$\begin{array}{l} \frac{dR}{d\beta }=\Sigma_{D}{\left(X^{t}|X^{t-\tau }\right)}^{-1}\\ -2\beta \Sigma_{D}{\left(X^{t}|X^{t-\tau }\right)}^{-1T}\Sigma_{D}\left(X^{t},X^{t-\tau }\right)\Sigma_{D}{\left(X^{t-\tau }\right)}^{-1}Q^{-1}\Sigma_{D}{\left(X^{t-\tau }\right)}^{-1}\Sigma_{D}{\left(X^{t},X^{t-\tau }\right)}^{T}\Sigma_{D}{\left(X^{t}|X^{t-\tau }\right)}^{-1}\\ -\beta^{2}\Sigma_{D}{\left(X^{t}|X^{t-\tau }\right)}^{-1T}\Sigma_{D}\left(X^{t},X^{t-\tau }\right)\Sigma_{D}{\left(X^{t-\tau }\right)}^{-1}\frac{dQ^{-1}}{d\beta }\Sigma_{D}{\left(X^{t-\tau }\right)}^{-1}\Sigma_{D}{\left(X^{t},X^{t-\tau }\right)}^{T}\Sigma_{D}{\left(X^{t}|X^{t-\tau }\right)}^{-1}, \end{array}$$ (30)

$$\frac{dQ}{d\beta }=\Sigma_{D}{\left(X^{t-\tau }\right)}^{-1}\Sigma_{D}{\left(X^{t},X^{t-\tau }\right)}^{T}\Sigma_{D}{\left(X^{t}|X^{t-\tau }\right)}^{-1}\Sigma_{D}\left(X^{t},X^{t-\tau }\right)\Sigma_{D}{\left(X^{t-\tau }\right)}^{-1},$$ (31)

and

$$\frac{dQ^{-1}}{d\beta }=-Q^{-1}\frac{dQ}{d\beta }Q^{-1},$$ (32)

$$=-Q^{-1}\Sigma_{D}{\left(X^{t-\tau }\right)}^{-1}\Sigma_{D}{\left(X^{t},X^{t-\tau }\right)}^{T}\Sigma_{D}{\left(X^{t}|X^{t-\tau }\right)}^{-1}\Sigma_{D}\left(X^{t},X^{t-\tau }\right)\Sigma_{D}{\left(X^{t-\tau }\right)}^{-1}Q^{-1}.$$ (33)

Inspection of the above equations reveals that dI*(*β*)/*dβ* = 0 is a quadratic equation with respect to *β*. Thus, *β* can be analytically computed without resorting to numerical optimization such as gradient ascent.

### Electrocorticogram (ECoG) recording

The detailed recording protocols were described in [38]. Here, we briefly describe the aspects of the protocols that are relevant for our analysis. We used customized multichannel ECoG electrode arrays. An array of ECoG electrodes was embedded in an insulating silicone sheet. The surface of the sheet was dimpled to expose the surface of ECoG electrodes with the diameter of 1 mm. The electrodes were made of platinum discs, and inter-electrode distance was 5 mm. We implanted 128 ECoG electrodes in the subdural space in four adult macaque monkeys. The ECoG electrodes covered the left hemisphere over the frontal, parietal, temporal, and occipital lobes. ECoG signal was recorded at a sampling rate of 1 kHz. All experimental and surgical procedures were performed in accordance with the protocols approved by the RIKEN ethics committee. During the experiments, the monkeys were seated in a primate chair with both arms and head restrained. We analyzed the data recorded when the monkeys were awake.

### Data processing and calculation of integrated information Φ*

To remove line noise and reduce artifacts in the ECoG data, we computed bipolar re-referenced signals between two neighboring electrodes. We calculated integrated information Φ* using all the bipolar re-referenced signals (64 in total). We considered the simplest partition scheme, “atomic partition” [39], in which the system is partitioned into its individual elements. For this data set, it meant that we computed Φ* assuming that all the 64 channels are independent. The atomic partition gives the upper bound of Φ* among all the possible partitions because it quantifies the amount of information loss when all the interactions in the system are ignored for decoding.

We approximated the probability distributions of the continuous ECoG signals with the Gaussian distribution. Under the Gaussian assumption, we analytically computed Φ* by using the equations derived in Methods. We estimated the covariance matrices of the data with a time window of 2s and a time step of 2s. Then, we averaged the covariance matrices over 600s and used the average of the covariance matrices for computation of Φ*.

## Supporting Information

S1 Text Mathematical details of integrated information. (PDF)

S1 Fig Theoretical requirements are not satisfied by previously proposed measures. (PDF)
