# Supplementary material for: Measuring Integrated Information from the Decoding Perspective
Source: PLoS Comput Biol. 2016 Jan 21;12(1):e1004654. doi: 10.1371/journal.pcbi.1004654 (PMC4721632; doi:10.1371/journal.pcbi.1004654)
Supplement: S1 Text — (PDF) [file pcbi.1004654.s001.pdf]

Measuring integrated information from the decoding perspective

Masafumi Oizumi[1,2,*], Shun-ichi Amari[1], Toru Yanagawa[1], Naotaka Fujii[1], Naotsugu Tsuchiya[2,3,*]
1 RIKEN Brain Science Institute, Wako, Saitama, Japan
2 School of Psychological Sciences, Faculty of Biomedical and Psychological Sciences, Monash University, Clayton, Victoria, Australia
3 Japan Science and Technology Agency, Kawaguchi, Saitama, Japan
∗ E-mail: oizumi@brain.riken.jp, naotsugu.tsuchiya@monash.edu

Supporting Information

A summary of intrinsic information, integrated information and mismatched decoding

In this section, we summarize three key concepts in this paper, namely, intrinsic information, integrated information and mismatched decoding. The former two concepts are introduced and explained in [1, 2]. The latter is explained in [3]. Here, we briefly summarize these concepts with somewhat different emphases than the original papers for readers who are not familiar with these concepts.

Intrinsic and extrinsic information

In IIT, information always refers to intrinsic information in contrast to extrinsic information [2]. Intrinsic and extrinsic here refers to the perspective from which information is considered. Intrinsic information is quantified from the perspective of the system itself while extrinsic information is quantified from the perspective of an external observer.

In neuroscience, the informational relationship between neural states X and external stimuli S (or observable output behaviors) have been extensively quantified [4–7], in a form of the mutual information I between X and S as

$$I(X;S) = H(S) - H(S|X). \tag{1}$$

where the entropy $H(S)$ and the conditional entropy $H(S|X)$ are given by

$$H(S) = -\sum_{s} p(s)\log p(s), \tag{2}$$

$$H(S|X) = -\sum_{x,s} p(s,x)\log p(s|x). \tag{3}$$

Here, x and s represent a particular neural state and a particular external stimulus, respectively, with $p(x)$, $p(s)$, $p(s,x)$, and $p(s|x)$ denoting the probability of x and s, the joint probability of x and s, and a conditional probability of s given x. The sum is calculated for all possible neural states x or over all stimuli s. The capital S and X represent an entire set of s or x, respectively (Replace the sum $\sum$ with the integral $\int$ when neural states are represented with continuous variables). As shown in Eq. 1, mutual information is expressed as the difference between the entropy of stimuli, $H(S)$, and the conditional entropy of stimuli given neural states, $H(S|X)$. Thus, $I(X;S)$ quantifies the reduction of uncertainty about stimuli by acquiring knowledge of neural states from the perspective of an external observer, i.e. to what extent can an external observer know about external stimuli by observing neural states. This type of information is called extrinsic information because the information is quantified from an external observer's point of view.

Intrinsic information, in contrast, should depend only on internal variables of the system and is quantified from the viewpoint of the system itself, independent of external variables, requiring no external

observers [2]. If information concerns consciousness, it should be intrinsic information, because consciousness is independent of external observers. With this concept of intrinsic information, IIT aims to quantify how much "difference" the internal mechanisms of a system makes for the system itself, i.e. the degree of influence a system exerts on itself through its internal causal mechanisms. How the past states would affect the present states can be determined by the transition probability matrix of the system, $p(X^t|X^{t-\tau})$, which specifies probabilities according to which any state of a system transits to any other state. Here, X^t and $X^{t-\tau}$ are the present and past states of the system at time t and $t-\tau$, respectively. IIT quantifies intrinsic information using the transition probability matrix.

The intrinsic information proposed in IIT 2.0 quantifies to what extent the mechanisms of the system make the posterior probability distribution of the past states given a present state different compared with a prior distribution of the past states. The posterior probability distribution of the past states given a present state represents the likelihood of potential causes of the given present state. Intrinsic information in IIT 2.0, which is called "effective information", is defined as the difference between the posterior probability distribution, $p(X^{t-\tau}|x^t)$, and a prior distribution of the past states, $p(X^{t-\tau})$ as follows:

$$ei(x^t) = D_{KL}\left(p(X^{t-\tau}|x^t)||p(X^{t-\tau})\right), \tag{4}$$

where $D_{KL}(p(X)||q(X))$ is the Kullback-Leibler divergence, which measures the distance between the two probability distributions p and q and is given by

$$D_{KL}(p(X)||q(X)) = \sum_x p(x)\log\frac{p(x)}{q(x)}. \tag{5}$$

If there are no causal mechanisms within the system, the present states are not affected by the past states. Thus, the posterior distribution of the past states does not differ from the prior distribution. IIT interprets the degree of the "difference" made in the posterior probability distribution of the past states according to its internal mechanisms, as information generated intrinsically within the system. Note that while intrinsic information is based on an intrinsic property of the system, it does not mean that it cannot be quantified by an external observer.

To quantify intrinsic information, in addition to the transition probability matrix, a prior distribution of the past states must be specified. Although the transition probability matrix is determined by the intrinsic mechanisms of a system, a prior distribution of the past states cannot be uniquely determined. There are many possible methods to choose a prior distribution from different standards. For example, in the context of channel capacity in information theory, the prior distribution that maximizes information may be selected [8]. In contrast, IIT selects the maximum entropy distribution as a prior distribution [1,9]. If a system's states are represented as a set of discrete variables, the maximum entropy distribution is the uniform distribution over all possible past states $X^{t-\tau}$. Thus, using the maximum entropy distribution as a prior distribution means that every possible past state is equally likely as a cause of a present state.

Although the maximum entropy distribution can be uniquely defined for discrete variables, this is not possible for continuous variables [8,10]. If some constraints are given, the maximum entropy distribution can be defined for continuous variables. For example, under the constraints that the mean and the variance of the variables are fixed at specific values, the Gaussian distribution with the specified mean and variance is the maximum entropy distribution. There is no principle that determines what types of constraints should be imposed on and how the maximum entropy distribution should be uniquely determined for continuous variables. Thus, intrinsic information (and integrated information) defined in IIT 2.0 can be applied only to discrete variables.

Using entropy, Eq. 4 can be written as

$$ei(x^t) = H(p({}^{\max}X^{t-\tau})) - H(p({}^{\max}X^{t-\tau}|x^t)), \tag{6}$$

where the superscript max placed on the left side of $X^{t-\tau}$ is a reminder that the distribution of $X^{t-\tau}$ is the maximum entropy distribution. Eq. 6 provides another interpretation of effective information. It

quantifies to what extent uncertainty of the past states $X^{t-\tau}$ (the entropy, $H(^{\max}X^{t-\tau})$) can be reduced by knowing a particular present state x^t from the system's intrinsic point of view. Using Bayes' rule, the posterior distribution, $p(^{\max}X^{t-\tau}|x^t)$, can be calculated as

$$p(^{\max}X^{t-\tau}|x^t) = \frac{p(x^t|^{\max}X^{t-\tau})p(^{\max}X^{t-\tau})}{p(x^t)}. \tag{7}$$

Averaging $ei(x^t)$ over all possible present states x^t, the averaged effective information equals the mutual information between the past and present states,

$$\begin{aligned} EI &= \sum_{x^t} p(x^t)ei(x^t), & (8) \\ &= H(p^{\max}(X^{t-\tau})) - H(p(^{\max}X^{t-\tau}|X^t)), & (9) \\ &= I(^{\max}X^{t-\tau}; X^t). & (10) \end{aligned}$$

While effective information is originally quantified in a state-dependent manner as in Eq. 4 (with a particular present state, x^t), we consider only the averaged effective information in Eq. 10 (with an entire set of present states, X^t) following the previous study [10].

Integrated information

Integrated information is the quantity that measures the information generated by the system as a whole above and beyond the information generated independently by its parts [1, 11]. As performed when computing intrinsic information, integrated information is computed between the system's past $X^{t-\tau}$ and present states X^t. Consider partitioning a system into m parts such as M_1, M_2, $\cdots$, and M_m and computing the amount of information that is integrated across m parts. Quantifying integrated information is equivalent to quantifying the amount of information lost by partitioning the system. In IIT, partitioning into m parts corresponds to splitting the transition probability matrix $p(X^t|X^{t-\tau})$ into the product of each transition probability matrix in the parts $p(M_i^t|M_i^{t-\tau})$. The partitioned transition probability matrix, $q(X^t|X^{t-\tau})$, can be written as

$$q(X^t|X^{t-\tau}) = \prod_{i=1}^{m} p(M_i^t|M_i^{t-\tau}). \tag{11}$$

Integrated information, $\phi(x^t)$, proposed in IIT 2.0 is defined as the difference between the posterior probability distribution of the past states given a present state in the intact system, $p(^{\max}X^{t-\tau}|x^t)$ and that in the "partitioned" system, $q(^{\max}X^{t-\tau}|x^t)$ is as follows:

$$\phi(x^t) = D_{KL}\left(p(^{\max}X^{t-\tau}|x^t)||q(^{\max}X^{t-\tau}|x^t)\right), \tag{12}$$

where D_{KL} is the Kullback-Leibler divergence defined in Eq. 5, and the past states are assumed as the maximum entropy distribution. $q(^{\max}X^{t-\tau}|x^t)$ is defined as follows:

$$q(^{\max}X^{t-\tau}|x^t) = \frac{q(x^t|^{\max}X^{t-\tau})q(^{\max}X^{t-\tau})}{q(x^t)}, \tag{13}$$

where $q(x^t) = \sum_{X^{t-\tau}} q(x^t|X^{t-\tau})q(^{\max}X^{t-\tau})$ and $q(^{\max}X^{t-\tau})$ is the maximum entropy distribution. Integrated information defined in Eq. 12 quantifies the difference in the posterior probability distribution of the past states given a present state, if the parts of the system are forced to be independent.

Although the original integrated information measure $\phi(x^t)$ is defined for a particular present state x^t, we consider only the average of $\phi(x^t)$ over all possible states as is performed for quantifying information in the previous section. The averaged integrated information Φ can be calculated as follows:

$$\Phi = \sum_{x^t} p(x^t)\phi(x^t), \tag{14}$$

$$= \sum_{x^t} p(x^t) D_{KL}\left(p({}^{\max}X^{t-\tau}|x^t)||q({}^{\max}X^{t-\tau}|x^t)\right), \tag{15}$$

$$= \sum_{x^t} p(x^t) \sum_{x^{t-\tau}} p({}^{\max}x^{t-\tau}|x^t) \log \frac{p({}^{\max}x^{t-\tau}|x^t)}{q({}^{\max}x^{t-\tau}|x^t)}. \tag{16}$$

Using Eq 11 and 13, we can write Φ in terms of entropy as follows:

$$\Phi = \sum_{i=1}^{m} H({}^{\max}M_i^{t-\tau}|M_i^t) - H({}^{\max}X^{t-\tau}|X^t). \tag{17}$$

As shown in Eq. 17, integrated information measures the difference between the uncertainty of the past states given the present states in the intact system and that in the partitioned system. The uncertainty of the partitioned system is always larger than that of the intact system and the increase in uncertainty corresponds to the loss of information caused by partitioning. We can rewrite Eq. 17 in terms of mutual information as follows:

$$\Phi = I({}^{\max}X^{t-\tau}; X^t) - \sum_{i=1}^{m} I({}^{\max}M_i^{t-\tau}; M_i^t), \tag{18}$$

where we use the fact that the entropy of the whole system $H({}^{\max}X^{t-\tau})$ is the same as the sum of the entropy of the subsystems $\sum_{i=1}^{m} H({}^{\max}M_i^{t-\tau})$ when the maximum entropy distribution is assumed.

Quantitative meaning of I and I^* in information theory

In this section, we briefly review the quantitative meaning of mutual information I in information theory and that of its extension to mismatched decoding I^*, which was developed by Merhav et al. [12] (see also [3, 8, 13]). Consider information transmission over a noisy channel $p(Y|X)$ where X is the input and Y is the output. For simplicity, assume that X and Y are both 0 or 1. (In the Results section, we consider the case where X and Y are the past and present states of a system, $X^{t-\tau}$ and X^t, respectively, and the states of a system are multidimensional variables but the arguments as described below are applicable to such cases.) The sender transmits a sequence of X with length N called a code word, $c = [X_1, X_2, \cdots, X_N]$, over the noisy channel. For binary inputs, there are 2^N possible code words, but the sender does not transmit them all. A set of the code words transmitted over the noisy channel is called a codebook. The codebook is shared between the sender and the receiver. The transmitted code word is disturbed by the noise that depends on $p(Y|X)$ and is changed to $c' = [Y_1, Y_2, \cdots, Y_N]$, where Y_i is the output of X_i. The job of the receiver is to infer (decode) which code word is sent from the received message c'. Consider the question as follows: For the receiver to decode the message "error-free" (more precisely, with an infinitesimally small error with limits of $N \to \infty$), how many code words can the sender transmit, or how many code words can the codebook contain?

Shannon's noisy channel coding theorem answers this question. According to the noisy channel coding theorem, the mutual information determines the upper limit of the number of code words that can be sent error-free over a noisy channel. We denote the maximal number of code words that can be sent error-free over the noisy channel by 2^{RN}, where R is called the information transfer rate and is less than or equal to 1. The information transfer rate R is given by the mutual information I between X and Y,

$$R = I(X; Y). \tag{19}$$

To achieve the maximal information transfer rate given by the mutual information, the receiver must optimally decode a message, which can be performed using the maximum likelihood estimation. The maximum likelihood estimation means choosing the code word c in the codebook that maximizes the likelihood $p(c'|c)$,

$$p(c'|c) = \prod_i p(Y_i|X_i). \tag{20}$$

Note that the optimal decoding scheme uses the actual probability distribution $p(Y|X)$. This type of decoding is called matched decoding, because the probability distribution used for decoding is matched with the actual probability distribution. If a mismatched probability distribution $q(Y|X)$ other than $p(Y|X)$ is used for decoding, the information transfer rate necessarily degrades. The information transfer rate R^* for a mismatched decoding is given by I^*,

$$R^* = I^*(X;Y). \tag{21}$$

As in matched decoding, decoding is performed using the maximum likelihood estimation with the following "mismatched" likelihood function $q(c'|c)$,

$$q(c'|c) = \prod_i q(Y_i|X_i). \tag{22}$$

$I^*(X;Y)$ is an extension of the mutual information $I(X;Y)$ in the sense of the information transfer rate over a noisy channel $p(Y|X)$ when a mismatched distribution $q(Y|X)$ is used for decoding.

The information transfer rate determines the amount of information that can be obtained from a message. The receiver obtains more information from a message when the information transfer rate increases. The mutual information I, which is equivalent to the maximal information transfer rate, determines the maximum amount of information that can be obtained by matched decoding. I^*, in contrast, determines the amount of information that can be obtained by a mismatched decoding.

Equivalence of Φ^* with Φ under the assumption of maximum entropy distribution

We show that Φ^* is equivalent to Φ, proposed by [1] when the maximum entropy distribution is assumed for the past state as follows.

First, we should note that when the maximum entropy distribution is assumed, the distribution of the past states in the whole system can be decomposed into the product of the distribution of each part as

$$p({}^{\max}X^{t-\tau}) = \prod_i p({}^{\max}M_i^{t-\tau}). \tag{23}$$

Bearing this in mind, we can compute I^* as follows

$$\begin{aligned} I^*(\beta) = &-\int dX^t p(X^t) \log \prod_i \int dM_i^{t-\tau} p({}^{\max}M_i^{t-\tau}) p(M_i^t|M_i^{t-\tau})^\beta \\ &+ \beta \int dX^{t-\tau} \int dX^t p({}^{\max}X^{t-\tau}) p(X^t|X^{t-\tau}) \log \prod_i p(M_i^t|M_i^{t-\tau}), \qquad (24) \\ &= -\sum_i \int dM_i^t p(M_i^t) \log \int dM_i^{t-\tau} p({}^{\max}M_i^{t-\tau}) p(M_i^t|M_i^{t-\tau})^\beta - \beta \sum_i H(M_i^t|M_i^{t-\tau}). \qquad (25) \end{aligned}$$

To obtain β that maximizes I^*, we differentiate $I^*(\beta)$ as

$$\frac{dI^*(\beta)}{d\beta} = -\sum_i \int dM_i^t \frac{p(M_i^t)}{r(M_i^t)} \frac{dr(M_i^t)}{d\beta} - \sum_i H(M_i^t|M_i^{t-\tau}). \tag{26}$$

where

$$r(M_i^t) = \int dM_i^{t-\tau} p({}^{\max}M_i^{t-\tau}) p(M_i^t|M_i^{t-\tau})^\beta, \tag{27}$$

$$\frac{dr(M_i^t)}{d\beta} = \int dM_i^{t-\tau} p({}^{\max}M_i^{t-\tau}) p(M_i^t|M_i^{t-\tau})^\beta \log p(M_i^t|M_i^{t-\tau}), \tag{28}$$

Substituting $\beta = 1$ into $\frac{dI^*(\beta)}{d\beta}$, we obtain

$$\frac{dI^*(\beta=1)}{d\beta} = -\sum_i \int dM_i^t \int dM_i^{t-\tau} p^{\max}(M_i^{t-\tau}) p(M_i^t|M_i^{t-\tau}) \log p(M_i^t|M_i^{t-\tau}) - \sum_i H(M_i^t|{}^{\max}M_i^{t-\tau}), \tag{29}$$

$$= 0, \tag{30}$$

We therefore find that $I^*(\beta)$ is maximized when $\beta = 1$. Substituting $\beta = 1$ into $I^*(\beta)$, we obtain the expression of I^* as

$$I^*(\beta=1) = \sum_i H(M_i^t) - \sum_i H(M_i^t|{}^{\max}M_i^{t-\tau}), \tag{31}$$

$$= \sum_i I({}^{\max}M_i^{t-\tau}; M_i^t). \tag{32}$$

From Eq. 32, we see that our measure is equivalent to the original measure when the maximum entropy distribution is used.

Φ^* is 0 when parts are perfectly correlated

We show that Φ^* is 0 when parts are perfectly correlated. For simplicity, we consider a system consisting of two units M_1 and M_2 and the mismatched decoder, $q(X^t|X^{t-\tau}) = p(M_1^t|M_1^{t-\tau})p(M_2^t|M_2^{t-\tau})$. It is easy to generalize to the case of more than two units. When the two units are perfectly correlated, $M_1^{t-\tau} = M_2^{t-\tau}$ and $M_1^t = M_2^t$. In this case, the joint probability distribution can be written as $p(X^{t-\tau}) = p(M_1^{t-\tau})p(M_2^{t-\tau}|M_1^{t-\tau}) = p(M_1^{t-\tau})\delta(M_2^{t-\tau} - M_1^{t-\tau})$ where $\delta(x)$ is the Dirac delta function. I^* can be calculated as follows.

$$I^*(\beta) = -\int dX^t p(M_1^t)\delta(M_2^t - M_1^t) \log \int dX^{t-\tau} p(M_1^{t-\tau})\delta(M_2^{t-\tau} - M_1^{t-\tau}) \prod_{i=1}^{2} p(M_i^t|M_i^{t-\tau})^\beta$$

$$+ \beta \int dX^{t-\tau} \int dX^t p(X^{t-\tau}, X^t) \log \prod_{i=1}^{2} p(M_i^t|M_i^{t-\tau}), \tag{33}$$

$$= -\int dM_1^t p(M_1^t) \log \int dM_1^{t-\tau} p(M_1^{t-\tau}) p(M_1^t|M_1^{t-\tau})^{2\beta} - 2\beta H(M_1^t|M_1^{t-\tau}). \tag{34}$$

To obtain β that maximizes I^*, we differentiate $I^*(\beta)$ as

$$\frac{dI^*(\beta)}{d\beta} = -\int dM_1^t \frac{p(M_1^t)}{r(M_1^t)} \frac{dr(M_1^t)}{d\beta} - 2H(M_1^t|M_1^{t-\tau}). \tag{35}$$

where

$$r(M_1^t) = \int dM_1^{t-\tau} p(M_1^{t-\tau}) p(M_1^t|M_1^{t-\tau})^{2\beta}, \tag{36}$$

$$\frac{dr(M_1^t)}{d\beta} = 2\int dM_1^{t-\tau} p(M_1^{t-\tau}) p(M_1^t|M_1^{t-\tau})^{2\beta} \log p(M_1^t|M_1^{t-\tau}), \tag{37}$$

Substituting $\beta = 1/2$ into $\frac{dI^*(\beta)}{d\beta}$, we obtain

$$\frac{dI^*(\beta=1/2)}{d\beta} = -2\int dM_1^t \int dM_1^{t-\tau} p(M_1^{t-\tau}) p(M_1^t|M_1^{t-\tau}) \log p(M_1^t|M_1^{t-\tau}) - 2H(M_1^t|M_1^{t-\tau}), \tag{38}$$

$$= 0, \tag{39}$$

We therefore find that $I^*(\beta)$ is maximized when $\beta = 1/2$. Substituting $\beta = 1/2$ into $I^*(\beta)$, we obtain the expression of I^* as

$$I^*(\beta = 1/2) = H(M_1^t) - H(M_1^t|M_1^{t-\tau}), \tag{40}$$

$$= I(M_1^{t-\tau}; M_1^t). \tag{41}$$

When M_1 and M_2 are perfectly correlated, mutual information in the whole system is just equal to the mutual information in each part, i.e., $I(X^{t-\tau}; X^t) = I(M_1^{t-\tau}; M_1^t) = I(M_2^{t-\tau}; M_2^t)$. Thus, Φ^* becomes 0.

$$\Phi^* = I(X^{t-\tau}; X^t) - I^*(X^{t-\tau}; X^t), \tag{42}$$

$$= I(M_1^{t-\tau}; M_1^t) - I(M_1^{t-\tau}; M_1^t), \tag{43}$$

$$= 0. \tag{44}$$

References

1. Balduzzi D, Tononi G (2008) Integrated information in discrete dynamical systems: Motivation and theoretical framework. *PLoS Comput Biol* **4**, e1000091.

2. Oizumi M, Albantakis L, Tononi G (2014) From the Phenomenology to the Mechanisms of Consciousness: Integrated Information Theory 3.0. *PLoS Comp Biol* **10**, e1003588.

3. Oizumi M, Ishii T, Ishibashi K, Hosoya T, Okada M (2010) Mismatched decoding in the brain. *J Neurosci* **30**, 4815-4826.

4. Rieke F, Warland D, de Ruyter van Steveninck R, Bialek W (1997) Spikes: exploring the neural code. (MIT Press, Cambridge, MA).

5. Dayan P, Abbott LF (2001) Theoretical Neuroscience. Computational and Mathematical Modeling of Neural Systems. (MIT Press, Cambridge, MA).

6. Averbeck BB, Latham PE, Pouget A (2006) Neural correlations, population coding and computation. *Nat Rev Neurosci* **7**, 358-366.

7. Quian Quiroga R, Panzeri S (2009) Extracting information from neuronal populations: information theory and decoding approaches. *Nat Rev Neurosci* **10**, 173-185.

8. Cover TM, Thomas JA (1991) Elements of information theory. New York: Wiley.

9. Jaynes ET (1957) Information theory and statistical mechanics. *Phys Rev* **106**, 620-630.

10. Barrett AB, Seth AK (2011) Practical measures of integrated information for time-series data. *PLoS Comput Biol* **7**, e1001052.

11. Tononi G (2008) Consciousness as integrated information: a provisional manifesto. *Biol Bull,* **215**, 216-242.

12. Merhav N, Kaplan G, Lapidoth A, Shamai Shitz S (1994) On information rates for mismatched decoders. *IEEE Trans Inform Theory* **40**, 1953-1967.

13. Latham PE, Nirenberg S (2005) Synergy, redundancy, and independence in population codes, revisited. *J Neurosci* **25**, 5195-5206.
