# Supplementary material for: Measuring Integrated Information from the Decoding Perspective
Source: PLoS Comput Biol. 2016 Jan 21;12(1):e1004654. doi: 10.1371/journal.pcbi.1004654 (PMC4721632; doi:10.1371/journal.pcbi.1004654)
Supplement: S1 Fig — (PDF) [file pcbi.1004654.s002.pdf]

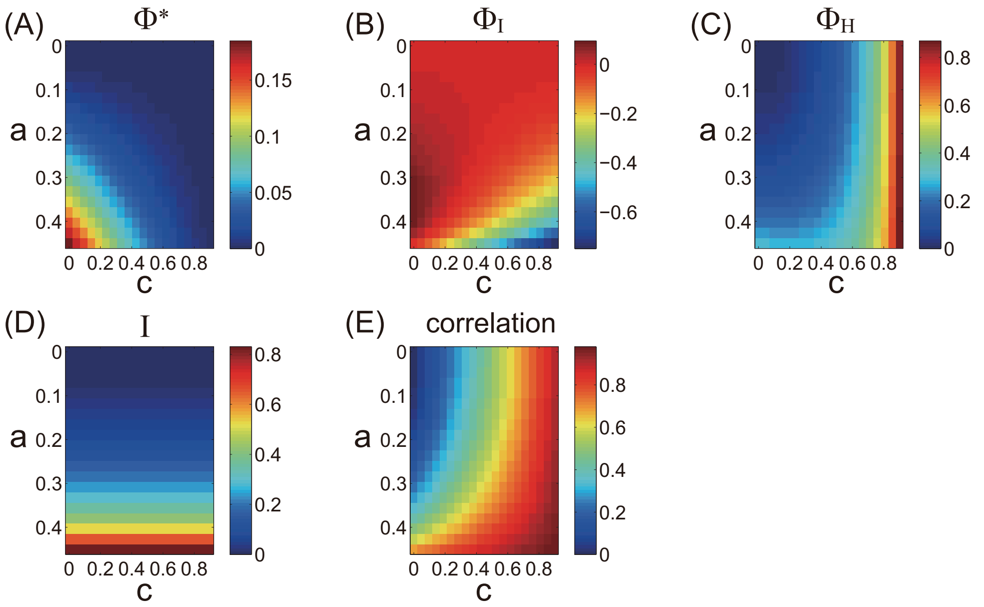

S1 Figure. Behaviors of Φ^* (A), Φ_I (B), Φ_H (C), mutual information I (D), and correlation (E) when the strength of connections a and the strength of noise correlation c are both varied in a linear regression model (Eq. 13 in the main text).

As we detailed in the main article, integrated information should be lower bounded by 0 and be upper bounded by the mutual information in the whole system I. In the main article, we used a simple linear regression model to demonstrate that Φ_I and Φ_H violate these bounds. Fig. S1 shows the behaviors of Φ^* (A), Φ_I (B), Φ_H (C), mutual information I (D), and correlation coefficient between units (E) when the strength of connections a and the strength of noise correlation c are both varied in the same linear regression model as in the main article (Eq. 13). As we can see in S1 Figure, Φ_I goes negative when the degree of correlation is high and thus, it does not satisfy the lower bound. Φ_H is not 0 even when there is no information ($a = 0$) and thus, it does not satisfy the upper bound. By comparing the panels (C) and (D), which are in the same color scale, we can see that Φ_H violates the upper bound when the noise correlation c is high. Φ^* always satisfies both the lower bound and the upper bound and therefore, it can be considered as a proper measure of integrated information.
